# Implementing a *Clostridium difficile* testing algorithm and its effect on isolation duration and treatment initiation: a pre- and post-implementation study

**DOI:** 10.1007/s10096-020-03823-w

**Published:** 2020-01-23

**Authors:** Erik Hans Vogelzang, Jacqueline Marleen Lankelma, Rosa van Mansfeld, Joffrey van Prehn, Robin van Houdt

**Affiliations:** 1 Department of Medical Microbiology and Infection Control, Amsterdam UMC, location VUmc, Amsterdam, the Netherlands; 2 Department of Medical Microbiology, Center for Infectious Diseases, Leiden University Medical Center, Leiden, the Netherlands

**Keywords:** *Clostridium difficile*, Isolation, Toxin A&B EIA and GDH test

## Abstract

A proportion of patients suspected of *Clostridium difficile* infection are unnecessarily placed in contact isolation. By introducing a random-access glutamate dehydrogenase (GDH) test for *C. difficile*, we aimed to reduce isolation time. In addition, we investigated whether the result of the toxin A&B enzyme immunoassay (EIA) was associated with the decision to initiate antibiotic treatment against *C. difficile*. This retrospective pre- and post-implementation study was from June 3, 2016, to June 4, 2018. Pre-implementation, only a NAAT was performed. In the post-implementation period, a GDH test was performed; if positive, a toxin A&B EIA followed the same day and subsequently a NAAT. Contact isolation for CDI was discontinued when the GDH test was negative. Median time in isolation was 50.8 h pre-implementation (*n* = 189) versus 28.0 h post-implementation (*n* = 119), *p* < 0.001. The GDH test had a negative predictive value of 98.8% (95% CI 97.9–99.4). In 7/31 (22.6%) patients with a positive NAAT and GDH test and a negative toxin A&B EIA, no antibiotics against *C*. *difficile* were initiated versus 4/28 (14.3%) patients who were NAAT, GDH and toxin A&B EIA positive. Introducing a random-access screening test resulted in a significant decrease in patient isolation time. The GDH test had a high negative predictive value making it suitable to determine whether contact isolation can be discontinued. Furthermore, the result of a toxin A&B EIA had limited added value on the percentage of patients in whom antibiotic treatment against *C. difficile* was initiated.

## Introduction

*Clostridium difficile*, recently reclassified as *Clostridioides difficile* [1], is the most common cause of nosocomial infectious diarrhoea in hospital patients [2]. Nosocomial outbreaks occur regularly, posing a risk for patients due to *C. difficile* infection (CDI) attributed mortality and morbidity [2, 3]. These outbreaks are associated with delayed diagnosis and absence of adequate infection prevention measures [4, 5].

To prevent nosocomial outbreaks of *C. difficile*, contact isolation is often imposed for patients suspected of CDI. However, some patients are placed in contact isolation unnecessarily. This leads to an increase in costs and workload (e.g. putting on gloves and gown when entering a patient’s room). A screening test for *C. difficile* could benefit patients, hospital workers and the hospital. Several (rapid screening) tests for *C. difficile* are available, such as a glutamate dehydrogenase (GDH) test and a toxin A&B enzyme immunoassay (EIA). However, testing for *C. difficile* is challenging from an infection prevention perspective; if the sensitivity of the testing strategy is too low, there is a risk of nosocomial transmission [6, 7]. If the testing strategy has low specificity, a substantial proportion of patients is placed in contact precautions unnecessarily.

Diagnosing CDI solely based on laboratory tests such as the GDH, toxin A&B EIA or nucleic acid amplification testing (NAAT) is challenging. A large study from 2015 illustrates that exclusive reliance on molecular tests for CDI without testing for toxins is likely to result in over-diagnosis [8]. Nevertheless, a toxin test has its downsides, since CDI-related complications may still occur in patients who are NAAT positive and toxin negative. In addition, the available toxin tests display large differences in diagnostic accuracy [9]. Besides, toxins are most likely detected if the stool specimen is tested immediately after collection since these toxins degrade quickly. However, in a hospital setting, a delay between faeces collection and testing occurs regularly.

There are different testing recommendations for CDI. The Infectious Diseases Society of America (IDSA) recommends algorithms in which a toxin A&B test is implemented [10]. Similar recommendations are made by the European Society of Clinical Microbiology and Infectious Diseases (ESCMID) [11].

Despite these recommendations, no optimal diagnostic algorithm exists to decide whether contact precautions should be continued or discontinued.

In this study, we describe the effect of implementing a rapid *C. difficile* screening test (GDH) in a random-access manner on isolation duration. Furthermore, we evaluated the diagnostic accuracy of the GDH test and the clinical value of a toxin A&B EIA in daily clinical practice based on antibiotic treatment initiation against *C. difficile*.

## Methods

### Study design

This retrospective pre- and post-implementation study was performed in a Dutch tertiary academic hospital. The pre-implementation period ran from June 3, 2016, till June 3, 2017. The post-implementation period was from June 4, 2017, to June 4, 2018. In the pre-implementation study period, only a NAAT for *C*. *difficile* was performed. In the post-implementation period, the testing algorithm consisted of a GDH test, performed immediately on arrival of a stool specimen at the hospital microbiology laboratory, followed by a NAAT. If the GDH test was positive, an additional toxin A&B EIA was performed on the same day. No tests were performed during the weekend.

In the pre-implementation period, contact precautions were (dis)continued based on the NAAT result. Post-implementation, contact precautions were discontinued when the GDH result was negative; these results were reported immediately to infection prevention staff. The hospital isolation policy stated that for patients who are highly suspected of CDI, contact precautions, consisting of a private room with a dedicated toilet and putting on gloves and a gown when entering a patient’s room, should be executed.

All samples tested according to the testing algorithm were included consecutively. The isolation duration was calculated for patients with a negative NAAT pre- and post-implementation of the testing algorithm. We regarded the NAAT as the most sensitive and specific test to detect *C. difficile*. We excluded patients with a positive NAAT from our isolation duration analysis since they could potentially have CDI. For these patients, isolation time is not reducible by introducing a random-access GDH test. For the same reason, we excluded patients with a positive norovirus test (for which the same contact precautions are imposed). Routine laboratory and clinical staff carried out the testing, ordering and clinical decision-making. We also investigated the diagnostic accuracy of the GDH test in routine clinical practice compared to the NAAT. Finally, we evaluated if a toxin A&B EIA had additional clinical value based on whether patients were treated for CDI.

### Microbiological procedures

Laboratory technicians performed the testing in the hospital microbiology laboratory according to local protocols and manufacturer’s instructions. In the post-implementation period, the stool specimen was tested immediately on arrival using the ImmunoCard® *Clostridium difficile* GDH (Meridian Bioscience Inc., Cincinnati, OH, USA). If the GDH test result was positive, the sample was further tested using an ImmunoCard® toxin A&B (Meridian Bioscience Inc., Cincinnati, OH, USA). Subsequently, all stool specimens were tested using NAAT. Stool was dissolved in 1 mL stool transport and recovery buffer (S.T.A.R.-buffer) (Roche Diagnostics GmbH, Mannheim, Germany) and kept at − 80 °C for at least 1 h. Following 10 min incubation at 100 °C, DNA was extracted using the MagNA-Pure96 platform (Roche Diagnostics GmbH, Mannheim, Germany). DNA was then amplified using a Real-Time PCR targeting the *C. difficile* toxin genes *cdtA* and *cdtB*, using the LightCycler480 platform (Roche Diagnostics GmbH, Mannheim, Germany), as previously described [12].

### Data collection and statistical analyses

Clinical data and isolation data was collected from the electronic medical records. The test results were collected from the laboratory information system. From the laboratory information system, we also extracted three important time intervals in testing for *C. difficile*: (1) from ordering the test by a physician till arrival of the stool specimen at the laboratory, (2) from arrival of the stool specimen at the laboratory till the test result, and (3) from ordering the test for *C. difficile* to the result reported to the clinic.

Isolation duration and time intervals were compared using a Mann-Whitney *U* test. A Pearson chi-square test was used to compare the difference in the proportion of patients in whom antibiotics against *C difficile* were initiated depending on the toxin A&B EIA test result. A *p* value of < 0.05 was considered statistically significant. The sensitivity, specificity, negative predictive value (NPV) and positive predictive value (PPV) of the GDH test were calculated and reported with 95% confidence intervals using the NAAT as a gold standard. All analyses were performed using IBM SPSS Statistics for Windows, version 22 (IBM Corp., Armonk, NY, USA). The 95% confidence intervals were calculated using R Programming (R Core Team 2018, R foundation for Statistical Computing, Vienna, Austria.)

## Results

### Samples

During the pre-implementation period, a total of 1451 test were ordered of which 1125 tests of 769 individual patients were included. For 326 orders, no sample arrived at the laboratory (22.5%). In the post-implementation period, a total of 1364 diagnostic tests were ordered, of which 1033 (75.7%) samples of 698 individual patients were included. For 305 orders (22.4%), no sample arrived at the laboratory; 23 (1.7%) samples were not included since the diagnostic algorithm was not followed; in 2 (0.1%) samples, the result of the NAAT could not be interpreted and 1 sample (0.1%) was no clinical isolate.

### Accuracy of GDH compared to NAAT

The sensitivity of the GDH test compared to the NAAT was 87.9% (95% CI 79.4–93.8). The NPV was 98.8% (95% CI 97.9–99.4) and PPV 65.0% (95% CI 55.9–73.4), see Table 1.

**Table 1:** Diagnostic accuracy of GDH compared with NAAT, *n* = 1033

Accuracy	GDH	95% CI
Sensitivity	87.9	79.4–93.8
Specificity	95.4	93.9–96.7
Positive predictive value	65.0	55.9–73.4
Negative predictive value	98.8	97.9–99.4

A total of 899 (87.0%) samples tested in the post-implementation period were NAAT and GDH negative. In 11/1033 (1.1%) samples of 10 patients, the GDH test was negative whereas the NAAT was positive. In three patients, antibiotics against CDI were initiated based on these test results (cycle threshold [Ct] values of the NAAT were 34.9, 40 and > 40, respectively). Seven samples, collected from 6 patients, had a Ct value ≥ 38.5 (one patient was tested twice after treatment initiation). The final patient sample had a Ct value of 37.8; in this patient, diarrhoea resolved spontaneously 2 days after the physician ordered testing for *C. difficile*.

In 43/1033 samples (4.2%), of 34 patients, GDH was positive and NAAT and toxin A&B EIA were negative. In five patients, treatment for *C. difficile* was initiated. In 2 patients, treatment was stopped after the treating physician was notified that the NAAT was negative; in two patients, treatment continued. The final patient in whom treatment for *C. difficile* was initiated had a positive GDH, NAAT and toxin A&B EIA test result 11 days before for which metronidazole was initiated. Based on the second GDH-positive result, the treating physician decided to initiate vancomycin treatment.

### Toxin A&B EIA in daily clinical practice

In 41/1033 samples (4.0%) of 31 individual patients, GDH and NAAT were positive and toxin A&B EIA was negative. In 7/31 (22.6%) patients, no antibiotics against *C. difficile* were initiated (Table 2). Of these 7 patients, 4 had no diarrhoea when the faeces sample was collected. In two patients, there was spontaneous resolution of the diarrhoea after the test results were known. One patient was diagnosed with *Salmonella* gastroenteritis.

**Table 2:** The number of individual patients in whom no treatment against *C. difficile* infection was initiated when the test results were known

Results*	No. of individual patients tested**	No. of individual patients in whom no treatment against *C. difficile* was initiated (%)
GDH negative, NAAT positive	10	7 (70.0)
GDH positive, NAAT negative, toxin A&B EIA negative	34	29 (85.3)
GDH positive, NAAT positive, toxin A&B EIA negative	31	7 (22.6)
GDH positive, NAAT positive, toxin A&B EIA positive	28	4 (14.3)

*No patients had a GDH-positive, NAAT-negative and EIA-positive result. The combination of a GDH-negative and toxin A&B EIA-positive result was not possible since the toxin A&B EIA was performed only if the GDH test was positive

**Some patients were tested multiple times

In 39/1033 samples (3.8%) belonging to 28 patients, GDH, NAAT and toxin A&B EIA were positive. In 4/28 (14.3%) patients, no antibiotic treatment was initiated. Two of these patients had no diarrhoea when faeces were collected. In one patient, there was spontaneous resolution of the diarrhoea. The final patient deceased before antibiotics could be initiated.

There was no significant difference between the number of patients with a negative or positive toxin A&B EIA in whom no treatment against *C. difficile* was initiated, respectively 7/31 (22.6%) vs. 4/28 (14.3%), *p* = 0.414.

### Isolation duration and processing time

Pre-implementation, the median time of isolation was 50.8 h (IQR 29.2–82.2) for patients who were NAAT negative and placed in isolation precaution (*n* = 189). After implementation of the GDH test, there was a significant reduction (*p* < 0.001) to a median time in isolation of 28.0 h (IQR 18.9–61.2, *n* = 119, see Table 3).

**Table 3:** Isolation duration for patients with a negative NAAT and time periods for the different important steps in testing for *C. difficile*

	Median hours (IQR)		
Time period	Pre-implementation, *n* = 189	Post-implementation, *n* = 119	*P* value
Isolation duration	50.8 (29.2–82.2)	28.0 (18.9–61.2)	< 0.001
From order to sample arrival at the laboratory	13.5 (2.2–20.4)	11.6 (2.8–18.1)	0.557
From sample arrival at the laboratory to test result	26.9 (22.8–29.9)	1.2 (0.8–2.2)	< 0.001
From order to final result reported to the hospital	32.9 (26.3–52.5)	17.7 (5.8–36.4)	< 0.001

The median time from arrival of the sample to first test result was significantly lower: 26.9 h (IQR 22.8–29.9) pre-implementation versus 1.2 h (IQR 0.8–2.2) post-implementation, *p* < 0.001. There was also a significant difference in the median time from order to result reported to the hospital: 32.9 h (IQR 26.3–52.5) pre-implementation versus 17.7 h (IQR 5.8–36.4) post-implementation, *p* < 0.001.

## Discussion

In our study, we found a significant reduction in unnecessary isolation for suspected CDI of approximately 23 h after implementing a random-access GDH test. The GDH test had a high negative predictive value (99%) making it suitable to determine whether contact isolation should be (dis)continued. Furthermore, a negative or positive toxin A&B EIA result in patients with a positive GDH and NAAT was not significantly associated with the decision to initiate treatment against *C. difficile* rendering addition of this test to an algorithm including a GDH and NAAT not expedient. Since contact isolation may lead to suboptimal care and increased costs, this reduction in isolation duration could potentially be beneficial for patients [13, 14].

A recent meta-analysis showed that the majority of patients admitted to the hospital are not colonized with *C. difficile* [15]. Therefore, using a test with high NPV such as the GDH test in a random-access manner to assess whether contact isolation should be continued or discontinued seems sensible. Our results are similar to those reported in several studies of diagnostic test accuracy in which the NPV varied between 97 and 100% [16–18]. Since the majority of patients tested negative for *C. difficile*, using a GDH test with a high NPV is a relatively inexpensive method compared to NAAT (e.g. GeneOhm assay, Cepheid Gene Xpert assay or in-house NAATs) for ruling out CDI. Although no assessment was made for cost-effectiveness, it is plausible that a reduction in isolation duration leads to a reduction in costs, since there is reduced workload and less personal protective equipment is needed [19].

The role of a toxin A&B EIA in the diagnostic algorithm remains unclear due to the reported low sensitivity [9], although a positive toxin A&B EIA and a positive GDH makes the diagnosis more likely as described in the IDSA guideline. However, our study result showed that in some patients, CDI is not present. In our study after clinical evaluation by the treating physician, clinical microbiologist, and sometimes an infectious disease specialist, there was no significant difference in treatment initiation against CDI for patients with a positive GDH and NAAT irrespective of the toxin A&B EIA result. Therefore, the toxin A&B EIA test result did not have a significant clinical impact. These results emphasize the unclear role of a toxin A&B EIA in diagnosing CDI in daily clinical practice, as previously described [20, 21]. Careful interpretation of laboratory tests, clinical signs, symptoms and risk factors (e.g., administration of multiple antibiotics, and recent hospitalization [22]) are necessary for diagnosing CDI.

Our study has some limitations. This study was based on faecal samples of patients, and some patients were tested multiple times. However, based on the high number of samples, the effect of repeated testing on the study results of patients is probably limited. Our study demonstrates that despite considerable reduction of the turnaround time, a significant proportion of the total time is from ordering till arrival of the sample which often cannot be influenced (i.e. time till the faeces can be collected from the patient). The actual time between faeces collection and arrival of the sample at the laboratory is probably lower than reported. However, this is applicable for both the pre- and post-implementation and therefore will not influence the results. The retrospective nature of this study potentially introduces bias since there is no strict study protocol and we are relying on others for recordkeeping. An advantage of our study is that it represents daily clinical practice. Furthermore, our study was performed in a single tertiary hospital, but when analysing the data, there was no indication that a specific subset of patients who only are admitted to a tertiary hospital were tested for *C. difficile*. Therefore, we believe these results are applicable for a general hospital.

In conclusion, our study demonstrates a reduction of unnecessary patient’s time in contact isolation by implementing a GDH test in a random-access manner, based on which isolation precautions were discontinued. Since contact isolation may lead to suboptimal care and increased costs, this is relevant for both patients and hospital staff. The GDH test had a high NPV making it suitable to determine whether contact isolation should be (dis)continued. Furthermore, the result of a toxin A&B EIA had only limited added value on the percentage of patients in whom antibiotic treatment against *C. difficile* was initiated. Therefore, the usefulness of testing for toxin A&B is questionable in daily clinical practice.

## Data Availability

The datasets used and/or analysed during the current study are available from the corresponding author on reasonable request.
